# Including People with Spinal Cord Injury in Research as Participants, Partners, and Personnel

**DOI:** 10.3390/ijerph20156466

**Published:** 2023-07-28

**Authors:** Kim D. Anderson

**Affiliations:** Department of Physical Medicine and Rehabilitation, MetroHealth System, School of Medicine, Case Western Reserve University, Cleveland, OH 44109, USA; kxa304@case.edu; Tel.: +1-216-957-3687

**Keywords:** rehabilitation medicine, people with disabilities, inclusion, underrepresented, spinal cord injury, national institutes of health

## Abstract

Individuals with disabilities are significantly underrepresented in research and are often not included in discussions on diversity, equity, inclusion, and accessibility. The Advisory Committee to the National Institutes of Health Director Working Group on Diversity formed an ad hoc Subgroup on Individuals with Disabilities to develop recommendations on how to enhance the inclusion of people with disabilities in the scientific workforce as well as throughout the research ecosystem. The article summarizes those recommendations and how they came about, then contextualizes them for the spinal cord injury (SCI) research field. Other fields that do not typically include individuals with disabilities in research can learn from the strong history of including people with SCI as research participants. There has been a growing drive within our field to enhance the inclusion of people living with SCI as research partners, but how are we doing with promoting their inclusion in the scientific workforce?

## 1. Introduction

People living with some form of disability make up 26% of the United States population [1] and represent 16% of the world population [2]. Despite this, individuals with disabilities experience significant societal barriers, health inequalities, and discrimination, all of which contribute to underrepresentation in multiple facets of the global human community.

To begin to address the underrepresentation of people with disabilities in science, in late 2021 a group of scientists living with disabilities, as well as key National Institutes of Health (NIH) staff, were pulled together by the Advisory Committee to the NIH Director (ACD) Working Group on Diversity. This Subgroup on Individuals with Disabilities was initially charged with creating a report that identified “*(1) Strategies to enhance data collection focused on individuals with disabilities in the scientific workforce, (2) Current data and trends on the prevalence of individuals with disabilities in the scientific workforce at various career stages, (3) Evidence-based practices for supporting individuals with disabilities, accounting for variation in disability type, (4) Programs with demonstrated success in supporting individuals with disabilities, and (5) Perspectives of individuals with disabilities*” [3]. In order to meet that charge, however, the subgroup realized it needed to be broadened to also include the health and healthcare disparities experienced by people with disabilities as well as their underrepresentation in research studies. The purpose of this communication is to provide a summary of the recommendations provided in a report that was endorsed by the ACD on 9 December 2022, and to contextualize some of those recommendations salient to the SCI research field.

## 2. Summary of Recommendations

To best understand the recommendations, it is strongly suggested to read the introductory core concepts and appendices of the report. The core concepts include an introduction to disability, language, health, and healthcare disparities experienced by people with disabilities, barriers faced by researchers with disabilities, intersectionality of disability, and universal design. The appendices contain disability data, disability definitions, and ableist language/beliefs examples, and a sampling of NIH-funded research projects demonstrating that health and healthcare disparities exist for people with disabilities. Below are the nine direct recommendations from the subgroup on individuals with disabilities [3].

1. *“Update the NIH mission statement”*—Removing the phrase “reducing disability” would shift the focus away from the medical model of disability belief that people with disabilities are flawed and need to be fixed.
2. *“Establish an NIH Office of Disability Research”*—This would be in line with other offices created for specific populations. Here, the purpose would be to advance strategies supporting the inclusion of people with disabilities as members of the scientific workforce as well as research participants and to advance disability-related research.
3. *“Establish an NIH Disability Equity and Access Coordinating Committee”*—This committee would serve as a resource for the Office of Disability Research and other diversity and equity efforts across NIH by seeking input from multiple disability groups in the community to suggest strategies for training and anti-ableism initiatives, how to best gather and report data on disability, and how to prevent discrimination and harassment from disability disclosure.
4. *“Develop an internal, NIH-wide effort to identify and address any structural ableism that may exist and promote disability inclusion by”*:
  - *“Fostering support for the equity, inclusion, and belonging of people with disabilities within NIH culture and structure”*—Similar efforts exist to address structural racism.
  - *“Advancing disability inclusion and anti-ableism through training, communication, policies, and accessibility”*—A multi-pronged approach is needed to overcome the pervasiveness of ableism.
5. *“Review policy, culture, and structure to identify opportunities to promote disability inclusion in the NIH-funded research workforce”*—NIH should lead the field by collecting and using data to develop evidence-based strategies to promote the best practices and programs that successfully promote the inclusion of people with disabilities in the scientific workforce.
6. *“Expand efforts to include disability communities and the perspectives of individuals with disabilities”*—Their input would help understand factors that perpetuate structural ableism, how to improve equity, inclusion, and access within NIH as well as across the external scientific community, how to increase disability cultural competency, as well as the experiences of researchers with disabilities with NIH grant application and review processes in a quantifiable manner.
7. *“Conduct research on disability health and health care disparities and equity by”*:
  - *“Formally designating people with disabilities as a health disparity population”*—Extensive evidence confirms that people with disabilities meet this legal designation, yet much is still needed to fully understand these health disparities and the factors that contribute to the inequities so that they can be overcome.
  - *“Funding and promoting research on health and health care disparities experienced by people with disabilities”*—This would also include analyzing grant applications and award data for potential barriers that contribute to the underfunding of health disparities research on people with disabilities.
  - *“Collecting data on disability wherever demographic information is collected within NIH data systems”*—This should be performed in collaboration with other Department of Health and Human Services (DHHS) partners to promote best practices in gathering and reporting disability data and to compare experiences of people with disabilities across the DHHS.
  - *“Supporting inclusion of disabled people as research participants”*—Although people with disabilities make up 26% of the US population, they are often excluded from research. Community engagement efforts should be made to actively recruit individuals with disabilities as research participants.
8. *“Ensure that disability inclusion and anti-ableism are core components of all NIH diversity, equity, inclusion, and accessibility (DEIA) efforts”*—Disability exclusion and ableism are structural issues that need to be addressed consistently across all NIH-wide DEIA efforts and should involve a senior advisor with lived experience of disability who has expertise in disability inclusion, equity, and research.
9. *“Maintain accountability for disability inclusion efforts”*—This should be performed through monitoring all the efforts described in the above recommendations and by sharing results publicly.

## 3. Contextualization for SCI Research Field

Although these recommendations were made in the context of NIH, we can all think about how to apply them in our research, clinical care, and the institutions that we work within. One way to be more thoughtful regarding inclusion is to think about the social model of disability in addition to the medical model of disability. The medical model of disability considers the disease or impairment to be the problem and the focus is on correcting the problem, i.e., correcting the flawed individual [4,5]. This is used very frequently in SCI research that focuses on topics such as limiting or repairing damaged spinal cord tissue, promoting neural plasticity, enhancing functional activities, or reducing secondary conditions. The social model of disability considers attitudes, the environment, and organizations as the problems which are preventing people with disabilities from fully participating in society [4,5]. Both models are important, but living in societies that are biased towards the able-bodied contributes significantly to the lack of inclusion of people with disabilities. To promote the full inclusion of everyone there is a need to focus on both the positive and negative attributes of the disease, the person, the environment, and society [6]. By challenging ourselves to think more broadly and be more intentional in inclusion, we can begin to lessen the impact of many barriers. Recommendations 5, 6, and 7 will be specifically discussed below with respect to the SCI field.

### 3.1. Inclusion of People with SCI as Research Participants

Research involving individuals living with SCI can provide examples to other research fields regarding how to effectively include people with disabilities in research as participants, particularly how to include people with physical disabilities, as there is a long history of successful inclusion. There are three main concepts in making research accessible: (1) universal design, (2) accommodation, and (3) modification [7,8]. Universal design involves designing a study from the beginning that enables all people to participate. For example, planning and getting approval for recruitment via a variety of outlets and response mechanisms that reach and are accessible to people with visual, hearing, or physical disabilities or ensuring that all materials needed by participants (consent forms, questionnaires, instructions) are available and approved in multiple formats (paper, electronic, auditory, visual) [7]. Accommodation involves changes that enable equal participation. Every participant may not need an accommodation(s), but when provided with accommodations, a participant is able to fully participate in a study. These may involve such accommodations as allowing extra time for completing tasks, scheduling visits later in the day, as mornings may be filled with self-care activities, or removing obstacles in cluttered areas to ease navigation through the research space [8]. Modification involves alterations to a standardized process, for example, modifying an intervention or assessment so a particular individual can participate. These should be performed with caution, as they have the potential to impact essential elements/constructs of what is being tested/measured [8]. If universal design and accommodations are employed, the need for modification should be greatly reduced. Many clinical SCI researchers have significant experience in design and accommodations that enable people with physical disabilities to participate in their research studies, but what about people with SCI that may also have vision or hearing impairment? We can all challenge ourselves to be more inclusive by employing universal design strategies further in our studies.

### 3.2. Inclusion of People with SCI as Research Partners

There has been a growing push in the SCI research field to include people living with SCI as meaningful partners throughout the research process. The five main steps in the process of research include (1) identifying the problem, (2) defining the question and obtaining funding, (3) collecting the data, (4) analyzing the data, and (5) disseminating the results. Significant strides have been made in obtaining input from people living with SCI in identifying the problems that need focus [9]. There are data demonstrating that the SCI community wants to be included throughout the full research cycle, however [10]. The ‘Integrated Knowledge Translation Guiding Principles for Conducting and Disseminating SCI Research in Partnership’ were published in 2020 as a set of guidelines to help researchers establish meaningful research partnerships throughout the research process and to do so in a manner that prevents tokenism [11]. Multiple funding agencies are requiring (Paralyzed Veterans of America Research Foundation, Department of Defense SCI Research Program, National Institute on Disability, Independent Living, and Rehabilitation Research) or encouraging (Craig H. Neilsen Foundation, National Institutes of Health) the inclusion of people with lived experience of SCI as partners with research grants. The idea is not to expect people with SCI to have scientific expertise, but rather to share their expertise of living with SCI to strengthen the research project. To that end, it is helpful for people with SCI to understand the research process at a high level to enable them to better advocate for the community as a whole in the research setting. The North American SC Consortium (NASCIC) released the SCI Research Advocacy Course in 2023. This is a free online course with 12 modules that focus on SCI research, the research process, and how to meaningfully engage as a research partner versus as a research participant [12].

### 3.3. Inclusion of People with SCI in the Scientific Workforce

The inclusion of people living with SCI in the scientific workforce is an area that is lagging behind significantly. Employment in general is very difficult for people with disabilities, for a myriad of complex interacting reasons and despite legal protections that exist and is even harder in the scientific fields [13]. There appears to be an increasing number of healthcare professionals with SCI [14,15], although there are no data thoroughly measuring this, and less is known about the number of individuals with SCI who are in the scientific workforce. The Craig H. Neilsen Foundation does have the Neilsen Scholarship Program specifically for individuals with SCI interested in pursuing higher education, both at the undergraduate and graduate level. The program does not restrict areas of focus and it is at seventeen select colleges, universities, and community colleges across the United States. The goal is to reduce socioeconomic barriers to successfully achieve a higher education. Although the program is not specifically driving people with SCI into the scientific workforce, it is specifically helping people with SCI gain education that can help them enter the workforce. Finally, employment opportunities for people with disabilities in general have recently expanded, primarily as a result of the COVID-19 pandemic [16,17]. In 2022 the employment rate for people with disabilities reached 21.3%, the highest since the Bureau of Labor Statistics began tracking these data in 2008 [18]. However, this is still significantly below the employment rates of people without disabilities. Some of the factors that have helped people with disabilities obtain employment since the pandemic include remote work options, flexible work hours, and job sharing [19].

## 4. Conclusions

Overall, persons with disabilities are significantly underrepresented in research. In the context of the SCI field, we can be an example to other fields for how to include individuals with disabilities as research participants, we are gaining momentum in developing partnerships with people with SCI throughout the research cycle, and we have a way to go toward enhancing the inclusion of people with SCI in the scientific workforce. Systemic and structural barriers, including accessibility and attitudinal barriers, need to be addressed to enhance inclusion of all kinds and we can each make a conscious effort in our daily lives to not perpetuate those barriers. Our field should continue to incorporate practices within our individual research laboratories and programs to promote diversity, equity, inclusion, and accessibility across the research ecosystem for individuals living with SCI.

## Data Availability

Not applicable.
